# Blood cholesterol-to-lymphocyte ratio as a novel prognostic marker to predict postoperative overall survival in patients with colorectal cancer

**DOI:** 10.1186/s12957-021-02471-4

**Published:** 2022-01-15

**Authors:** Siyu Zhou, Qian He, Nengquan Sheng, Jianfeng Gong, Jiazi Ren, Zhigang Wang

**Affiliations:** 1 Department of Gastrointestinal Surgery, Shanghai Jiao Tong University Affiliated Sixth People’s Hospital, No. 600 Yishan Road, Shanghai, 200233 China; 2 Shanghai Jiao Tong University School of Medicine, Shanghai, China

**Keywords:** Colorectal cancer, Cholesterol-to-lymphocyte ratio, Prognostic, nomogram

## Abstract

**Background:**

Lipid disequilibrium and systemic inflammation are reported to correlate with tumorigenesis and development of colorectal cancer (CRC). We construct the novel biomarker cholesterol-to-lymphocyte ratio (CLR) to reflect the synergistic effect of cholesterol metabolism and inflammation on CRC outcomes. This study aims to investigate the clinical significance of CLR and establish a prognostic model for CRC.

**Methods:**

Our study retrospectively enrolled 223 CRC patients who underwent curative surgical resection. The Kaplan-Meier method was employed to estimate the overall survival (OS) rates, and the association between serological biomarkers and survival was assessed with a log-rank test. Cox proportional hazard regression was applied in the univariate and multivariate analyses to identify independent prognostic factors, which were then used to develop a predictive nomogram model for OS in CRC. The nomogram was evaluated by the *C*-index, receiver operator characteristic curve (ROC) analysis, and calibration plot. All cases were grouped into three stratifications according to the total risk points calculated from the nomogram, and the difference in OS between them was assessed with the Kaplan-Meier method.

**Results:**

At the end of the study, death occurred in 47 (21%) cases. Patients with low CLR (< 3.23) had significantly prolonged survival (*P* < 0.001). Multivariate analyses revealed that N stage (*P* < 0.001), harvested lymph nodes (*P* = 0.021), and CLR (*P* = 0.005) were independent prognostic factors for OS and a prognostic nomogram was established based on these variables. The nomogram showed good calibration and predictive performance with a superior *C*-index than TNM stage (0.755 (0.719–0.791) vs. 0.663 (0.629–0.697), *P* = 0.001). Patients of different risk stratifications based on the total score of nomogram showed distinct survival (*P* < 0.001).

**Conclusions:**

The nomogram based on CLR and other clinical features can be used as a potentially convenient and reliable tool in predicting survival in patients with CRC.

**Supplementary Information:**

The online version contains supplementary material available at 10.1186/s12957-021-02471-4.

## Introduction

Colorectal cancer (CRC) is one of the most commonly diagnosed cancer and caused almost 900,000 deaths in 2019 [1, 2]. Despite advances in surgical treatment and chemoradiotherapy, the long-term outcome of CRC patients is still not optimistic [3, 4]. The prognosis of CRC is principally related to the tumor stage at diagnosis [5]. Additionally, several clinicopathological factors and serological indicators have been identified as predictors for survival in patients with CRC. Positive perineural invasion, poor differentiation, and elevation in preoperative carcinoembryonic antigen (CEA) levels are found associated with worse outcomes of CRC [6, 7]. However, the predictive performance of these factors remains unsatisfactory due to the high heterogeneity of the disease. Therefore, novel prognostic indicators and models are needed to improve the identification of patients who have a higher risk of death, so that appropriate treatment can be planned in advance.

A distinguishing feature of cancer cells is abnormal metabolism including lipid metabolism [8]. For instance, the transcription factor RORγ was found to be up-regulated and activate the cholesterol biosynthesis in triple-negative breast cancer cells [9]. Additionally, the uptake and the accumulation of cholesterol increase in CRC cells which promote their proliferation, and cancer occurrence and development seem to be associated with the consumption of circulating cholesterol by the tumor [10–12]. Therefore, hypercholesteremia potentially maintains sufficient cholesterol uptaken by tumors that accelerate the growth. Some studies report that serum lipid markers, such as total cholesterol, high-density lipoprotein cholesterol (HDL-C), and triglycerides, correlate with the prognosis of hepatocellular carcinoma and colorectal cancer [13–16]. In addition, the systemic inflammatory response also plays a vital role in cancer occurrence and progression, since cancer-associated inflammatory mediators aid in lymphovascular invasion, suppression for immune effector cells, and immune escape [17–19]. Several immune and systemic inflammation-associated biomarkers have been reported to be associated with long-term prognosis and postoperative complications such as anastomotic leakage in CRC [20–24]. Among these biomarkers, the lymphocyte counts, Glasgow prognostic score (GPS), modified GPS (mGPS), neutrophil-to-lymphocyte ratio (NLR), lymphocyte-to-monocyte-ratio (LMR), platelet-to-lymphocyte ratio (PLR), and C-reactive protein to albumin ratio were commonly used to predict CRC outcomes or therapeutic response [19, 25–32]. Furthermore, recent studies show that cholesterol accumulation and the metabolites of cholesterol in the tumor microenvironment (TME) have a positive impact on immunosuppression and pro-inflammation [9]. However, although one previous study focused on the prognostic value of HDL-C and the relationship between HDL-C and the immune signatures, it did not evaluate the combined effect of cholesterol metabolism and inflammation biomarkers on the prognosis of patients with CRC [33]. Therefore, the present study aims to assess the prognostic role of cholesterol-to-lymphocyte ratio (CLR) in CRC patients and develop and validate a novel prognostic model to provide a more precise risk stratification. Our findings may serve as a supplement to the TNM staging system and optimize clinical treatment decisions.

## Materials and methods

### Patient population

This retrospective study enrolled CRC patients who underwent curative surgical resection in the Department of General Surgery, Shanghai Jiao Tong University Affiliated Sixth People’s Hospital between June 2009 and December 2018. Participants were included based on the criteria below: (1) patients with pathologically confirmed primary CRC; (2) patients who received radical excision (R0); (3) no neoadjuvant chemoradiotherapy prior to the surgery. And cases with the following conditions were excluded: (1) metastatic disease; (2) incomplete clinicopathological data; (3) loss to follow-up; (4) history of other malignant tumors; (5) overall survival less than 1 month. This study was performed in agreement with the principles of the 1964 Helsinki Declaration, and approval from the ethics committee of the hospital was obtained (approval number: 2021-112). All involved patients were informed and signed an informed consent form.

### Data collection

Detailed clinical data were documented and collected from the medical record system, which comprised age, sex, tumor size, pTNM stage, tumor location, histological type, differentiation, lymphovascular invasion, perineural invasion, harvested lymph nodes number (LNs), CEA, CA199, lymphocyte count, neutrophil count, blood cholesterol and triglyceride. Blood routine examination, biochemical test, and analysis of serum tumor markers were performed within 7 days before the surgery. Tumor staging for CRC was according to the 8th edition of AJCC classification guidelines [34]. NLR was calculated as dividing neutrophil count by lymphocyte count, in accordance with previous literature [26, 35]. And CLR was calculated as dividing total serum cholesterol by lymphocyte count.

Patients were postoperatively treated in accordance with the national guidelines and were followed up regularly via telephone interviews. An independent researcher was involved in postoperative follow-up and data collection process. Follow-up was performed every 3 months after hospital discharge, and the survival status and the dates of death and telephone interview were recorded. All follow-ups ended in March 2021. The primary endpoint of this study was overall survival (OS), which was determined as the time from operation to death or the last follow-up.

### Statistical analysis

Numerical variables were shown as mean and standard deviation and categorical variables were presented in the form of numbers and percentages. Cholesterol and triglyceride were categorized by the upper reference limit (cholesterol: 5.9 mmol/L; triglyceride: 1.8 mmol/L) into the elevated and normal levels. NLR and CLR were classified as low and high levels based on the optimal cut-off points which were determined by the X-tile software.

The Kaplan-Meier method and log-rank test were used to investigate the correlation between clinical features and OS. We performed the univariate analysis with Cox proportional hazards regression to screen out the potential prognostic factors. Only variables with a *P* value less than 0.10 were further tested in the multivariate analyses. All Cox regression models in this study met the proportional hazard assumption. The significant factors in the multivariate analysis were then used to construct the nomogram which predicted the 3-year and 5-year OS rate of CRC patients. Using *rmda* package in R, a web-based dynamic calculator for the risk of death was built. Subgroup analysis for the prognostic value of the nomogram score was conducted and presented in the form of a forest plot. The concordance index (*C*-index) and calibration curve with 1000 bootstrap replications were applied to evaluate the prediction accuracy of the nomogram. Receiver operating characteristic (ROC) analyses were then performed, and the areas under the curves (AUC) were calculated to evaluate and compare the predictive performance of the nomogram, the non-CLR nomogram, and the TNM stage in the prediction for 3-year and 5-year OS. According to the total risk points calculated from the nomogram, patients were categorized into high, medium, and low-risk stratifications using the cut-off of 50% and 85% percentiles, and then KM analysis was performed to compare their survival difference.

All statistical analyses were conducted using R software (version 4.0.3 ). All tests were bilateral and variables with *P* < 0.05 were considered statistically significant.

## Results

### Patient characteristics

A total of 223 patients with CRC cancer were eligible and involved in this study, of which 134 (60%) patients were male and the other 89 (40%) were female. There were 67 patients (30%) below the age of 65 years, while 156 patients (70%) were older. Clinical stages I, II, and III were observed in 25 (11 %), 100 (45%), and 98 (44%) of the patients, respectively. Most (93%) of the patients were histologically diagnosed with adenocarcinoma, and 7% of the patients had other histological types of CRC. The cut-off values for NLR and CLR calculated by X-tile were 4.89 and 3.23, respectively. There were 134 (60%) patients with a low level of CLR (< 3.23). The median follow-up duration was 39 (range, 3–98) months. At the end of the study, death occurred in 47 (21%) cases (Table 1).

**Table 1:** Patient characteristics

Characteristics	Patients (*n* = 223)
Gender	
Male	134 (60%)
Female	89 (40%)
Age	
< 65	67 (30%)
≥ 65	156 (70%)
Size	
< 5 cm	135 (61%)
≥ 5 cm	88 (39%)
Tumor location	
Left colon	102 (46%)
Right colon	66 (30%)
Rectum	55 (25%)
Histological type	
Adenocarcinoma	207 (93%)
Others	16 (7%)
Differentiation	
Well/moderate	152 (68%)
Poor	71 (32%)
Lymphovascular invasion	
Negative	115 (52%)
Positive	108 (48%)
Perineural invasion	
Negative	40 (18%)
Positive	183 (82%)
Harvested LNs	
< 12	82 (37%)
≥ 12	141 (63%)
pT category	
T1, T2	35 (16%)
T3	77 (35%)
T4	111 (50%)
pN category	
N0	125 (56%)
N1	67 (30%)
N2	31 (14%)
pTNM stage	
Stage I	25 (11%)
Stage II	100 (45%)
Stage III	98 (44%)
CEA	
Negative	132 (59%)
Positive	91 (41%)
CA199	
Negative	179 (80%)
Positive	44 (20%)
Cholesterol	
Low	20 (9%)
High	203 (91%)
Triglyceride	
Low	182 (82%)
High	41 (18%)
NLR	
Low	193 (86%)
High	30 (14%)
CLR	
Low	134 (60%)
High	89 (40%)
Recurrence	
No	167 (75%)
Yes	56 (25%)
Survival	
Alive	176 (79%)
Dead	47 (21%)

*Abbreviations*: *LNs* Lymph nodes, *CEA* Carcinoembryonic antigen, *NLR* Neutrophil-to-lymphocyte ratio; *CLR* Cholesterol-to-lymphocyte ratio

### The association between clinical factors and OS

**Fig. 1:**
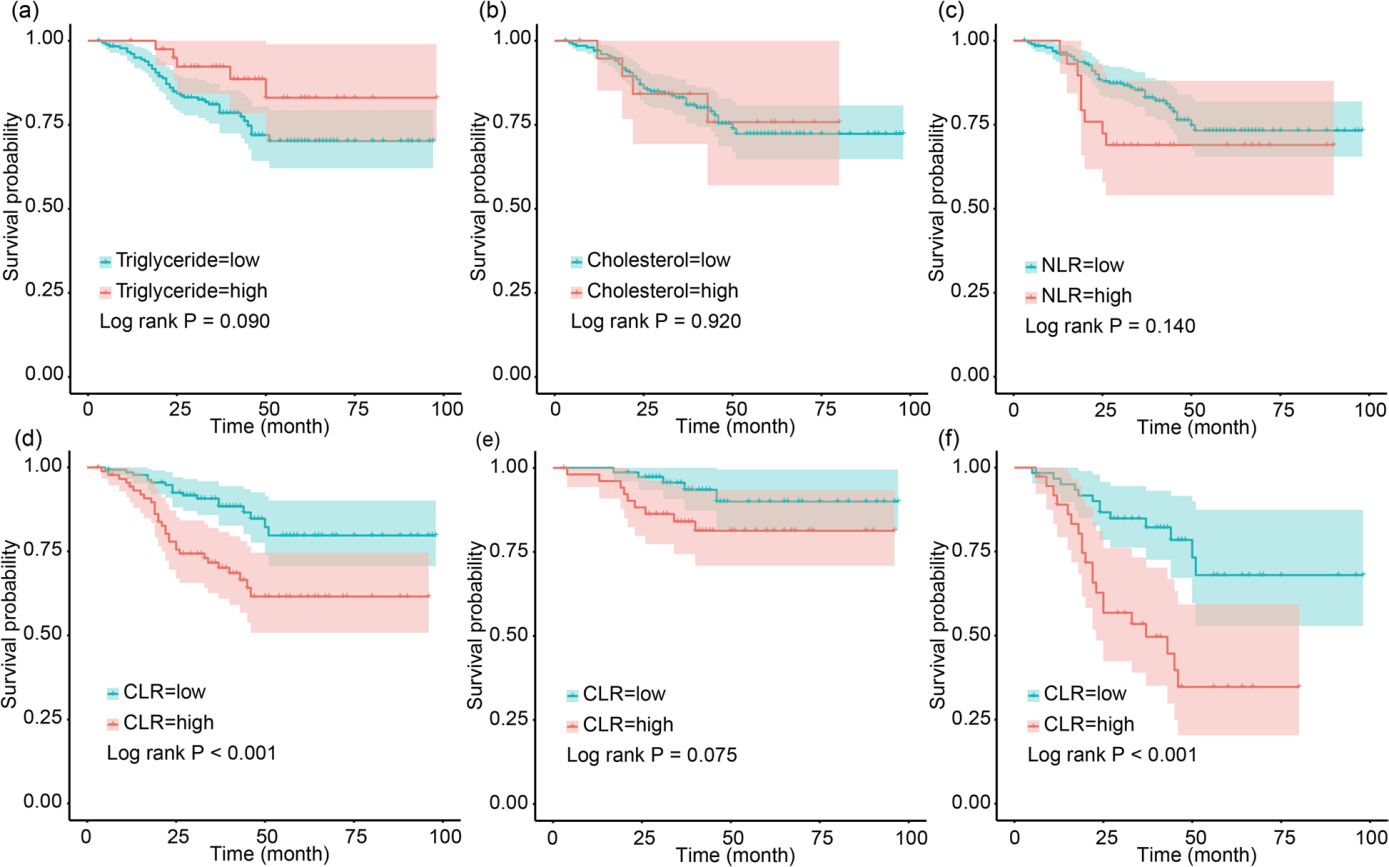
Kaplan-Mier survival curves for overall survival (OS) in colorectal cancer (CRC) patients stratified by **a** triglyceride, **b** cholesterol, **c** neutrophil-to-lymphocyte ratio (NLR), and **d** cholesterol-to-lymphocyte ratio (CLR). **e**, **f** Survival curves according to CLR levels in stages I and II and stage III CRC patients, respectively

Based on the Kaplan-Meier curves (Fig. 1), our results revealed that blood cholesterol (*P* = 0.920), triglyceride (*P* = 0.090), and NLR (*P* = 0.140) were not significantly associated with OS. However, patients with low CLR had more favorable OS than patients with high CLR (*P* < 0.001), with an improved 3-year OS rate (0.907 (95% CI: 0.858–0.959) vs. 0.716 (95% CI: 0.626–0.820)). In the subgroup analysis, CLR was prognostic for survival in clinical stage III (*P* < 0.001), but not in stages I and II (*P* = 0.075).

### Prognostic factors in the univariate and multivariate analyses

Univariate and multivariate analyses using Cox hazard proportion regression were applied to investigate the effect of clinicopathological characteristics on survival. Results of the univariate analysis revealed that N stage (N1 vs. N0, *P* < 0.001; N2 vs N0 (*P* = 0.001), lymphovascular invasion (*P* = 0.022), and CLR (*P* = 0.001) were significantly (*P* < 0.05) associated with the OS of CRC patients (Table 2). Together with these factors, to explore more potential prognostic variables, factors with P over 0.05 but less than 0.10 were also analyzed in the multivariate analysis.

**Table 2:** Univariate and multivariate Cox proportion hazard regression analysis for OS

Factors	Univariate analysis		Multivariate analysis	
	HR (95% CI)	*P* value	HR (95% CI)	*P* value
Age (≥ 65 vs. < 65)	1.309 (0.679–2.522)	0.421		
Gender (male vs. female)	1.298 (0.709–2.373)	0.397		
Tumor location (left colon vs. rectum)	0.586 (0.279–1.233)	0.159		
Tumor location (right colon vs. rectum)	1.267 (0.625–2.568)	0.511		
Size (≥ 5 cm vs. < 5cm)	1.300 (0.731–2.310)	0.371		
Histological type (others vs. adenocarcinoma)	0.733 (0.227–2.364)	0.604		
Differentiation (poor vs.well/moderate)	1.569 (0.875–2.811)	0.130		
Lymphovascular invasion (positive vs. negative)	2.006 (1.106–3.637)	0.022	0.865 (0.427–1.751)	0.687
Perineural invasion (positive vs. negative)	2.354 (0.844–6.557)	0.102		
Harvested LNs	0.960 (0.917–1.003)	0.073	0.945 (0.909–0.992)	0.021
pT category (T4 vs. T1–3)	1.316 (0.732–2.364)	0.358		
pN category				
N0	Ref.		Ref.	
N1	3.422 (1.750–6.690)	< 0.001	2.500 (1.179–5.302)	0.017
N2	3.960 (1.794–8.739)	< 0.001	5.115 (2.236–11.701)	< 0.001
CEA (positive vs. negative)	1.310 (0.738–2.324)	0.355		
CA199 (positive vs. negative)	0.845 (0.394–1.807)	0.664		
Cholesterol (high vs. low)	0.947 (0.339–2.639)	0.917		
Triglyceride (high vs. low)	0.457 (0.180–1.155)	0.098	0.495 (0.176–1.390)	0.181
NLR (high vs. low)	1.730 (0.835–3.578)	0.140		
CLR (high vs. low)	2.702 (1.500–4.867)	< 0.001	2.142 (1.254–3.659)	0.005

*Abbreviations*: *OS* Overall survival, *LNs* Lymph nodes, *CEA* Carcinoembryonic antigen, *NLR* Neutrophil-to-lymphocyte ratio, *CLR* Cholesterol-to-lymphocyte ratio

Consequently, the results of the multivariate analysis showed that N stage (N1 vs. N0, HR: 2.500 (1.179–5.302), P =0.017; N2 vs. N0, HR: 5.115 (2.236–11.701), *P* < 0.001), harvested LNs (HR: 0.945 (0.909–0.992); *P* = 0.021) and CLR (HR: 2.142 (1.254–3.659), *P* = 0.005) were identified as significant independent predictors for OS.

### Construction of the prognostic nomogram

**Fig. 2:**
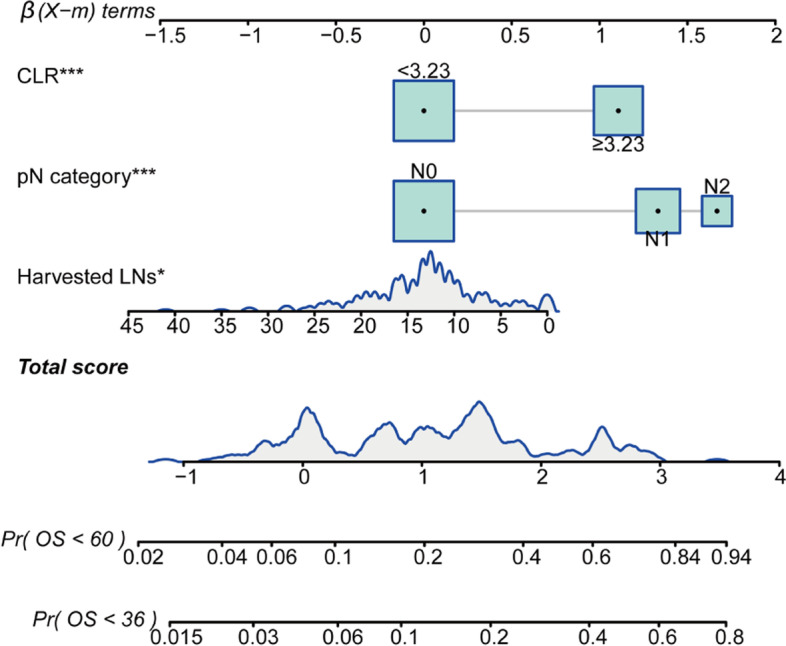
Nomogram for predicting overall survival (OS) rate at 3 and 5 years in patients with colorectal cancer (CRC). The variable score can be calculated by drawing a vertical line linking the value of each parameter with the *β*(*X* − *m*) terms at the top of this nomogram. Next, all scores are summed to obtain the total points score, which is plotted along the total points line, based on which the corresponding OS rates at 3 years and 5 years were obtained. CLR, cholesterol-to-lymphocyte ratio; LNs, lymph nodes; Pr, probability

According to the multivariate analysis, a nomogram that incorporated the independent prognostic factors including the N stage, harvested LNs, and CLR was constructed to predict the OS of CRC patients (Fig. 2 and Supplementary Table 1). According to the point scale bar, each of these variables was assigned with a coefficient. A total risk score was obtained from the nomogram and was used to assess the prognosis of the patient. Furthermore, to facilitate the calculation process, the nomogram was transformed into an online calculator with free access (https://zhousiyu.shinyapps.io/CLRNomogram/). By inputting clinical features and selecting a time point, the users could easily obtain the corresponding predicted survival rate.

### Nomogram validation

According to the subgroup analysis, our nomogram was prognostic for CRC regardless of the gender, age, tumor location, degree of differentiation, and pTNM stage (Supplementary Fig. 1). *C*-index was exploited to compare the predictive ability between the nomogram, non-CLR nomogram, and TNM stage (Table 3). The *C*-index of the nomogram was 0.755 (95% CI, 0.719–0.791), which indicated a good predictive accuracy for OS of patients with CRC. Furthermore, it was demonstrated that the nomogram had superior performance compared to TNM staging system (*C*-index: 0.755 (0.719–0.791) vs. 0.663 (0.629–0.697), *P* = 0.001). In addition, by comparing the nomogram with the non-CLR nomogram, it was indicated that the predictive performance could be significantly improved by adding CLR into the nomogram (*C*-index: 0.755 (0.719–0.791) vs. 0.703 (0.644-0.742), *P* = 0.020).

**Table 3:** *C*-indexes of prognostic factors or models for predicting OS

Factors or models	*C*-index	95% CI	*P* value
NLR	0.551	0.519–0.583	
CLR	0.636	0.600–0.672	
Nomogram	0.755	0.719–0.791	
Non-CLR nomogram	0.703	0.644–0.742	
TNM stage	0.663	0.629–0.697	
CLR vs. NLR			0.023
Nomogram vs. non-CLR nomogram			0.001
Nomogram vs. TNM stage			0.020

*Abbreviations*: *C-index*, concordance index; *OS*, overall survival; *CI*, confidence interval; *NLR*, neutrophil-to-lymphocyte ratio; *CLR*, cholesterol-to-lymphocyte ratio

Nomogram: CLR + harvested lymph nodes (LNs) + N stage. Non-CLR nomogram: harvested LNs + N stage

**Fig. 3:**
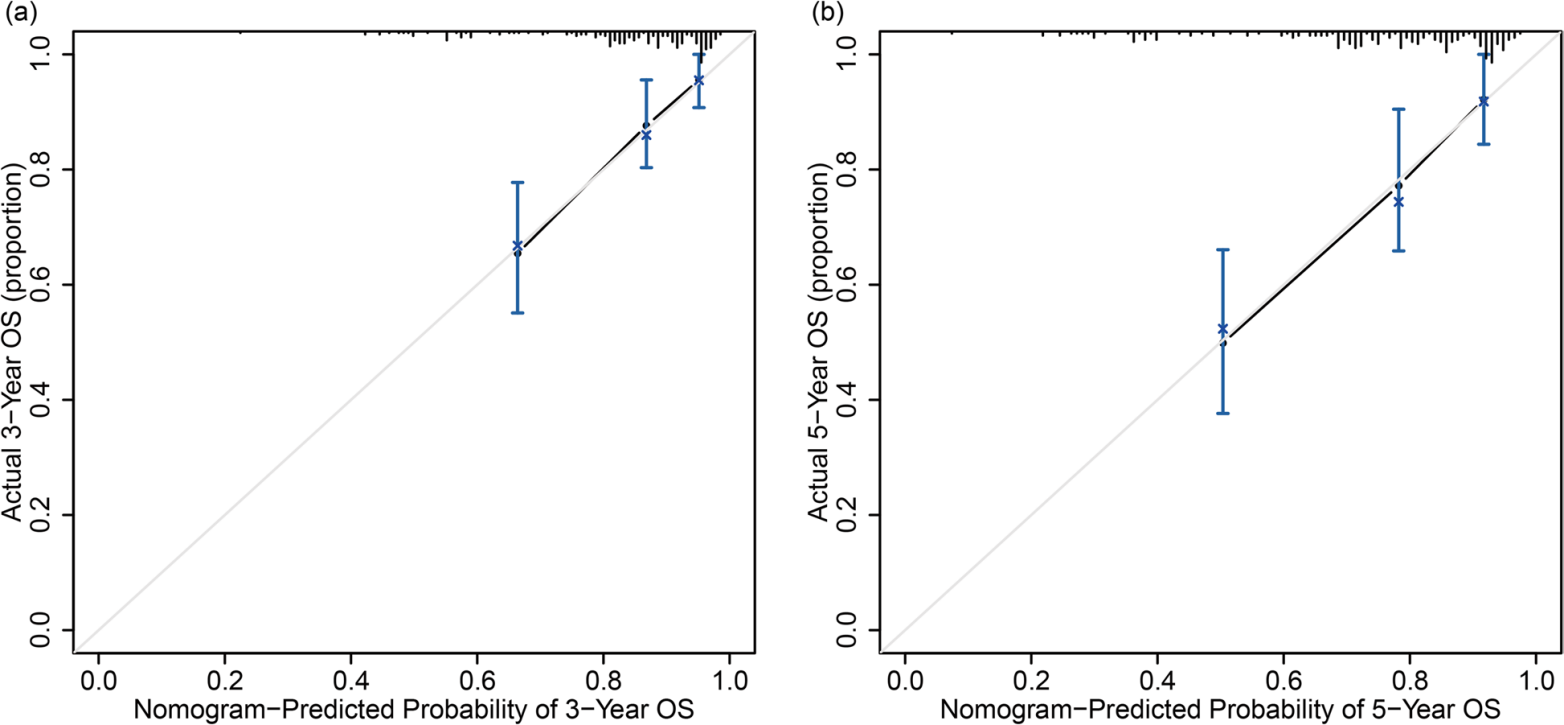
Calibration curves of the prognostic nomogram for predicting **a** 3-year and **b** 5-year overall survival (OS) in patients with colorectal cancer (CRC). The *Y*-axis represents the actual overall survival; the *X*-axis represents the nomogram-predicted overall survival. Each black point represents one-third of the total samples

**Fig. 4:**
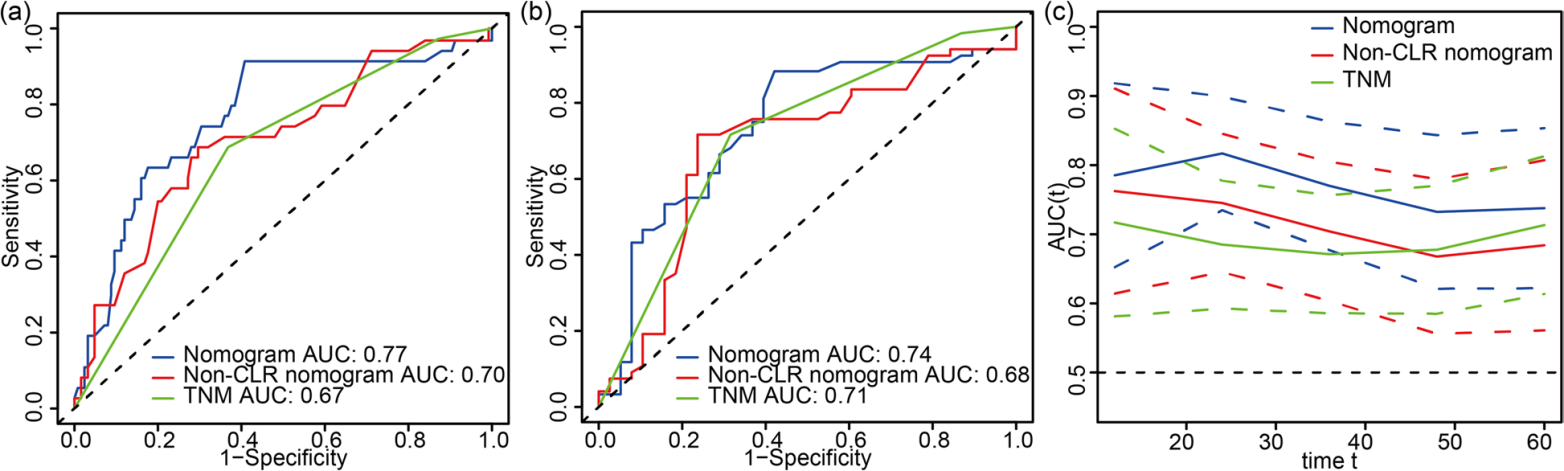
Receiver operator characteristic (ROC) analysis of the nomogram, non-CLR nomogram, and TNM stage for predicting survival at **a** 3 years and **b** 5 years, and **c** the area under ROC curve of the three models. CLR, cholesterol-to-lymphocyte ratio. Nomogram: CLR + harvested lymph nodes (LNs) + N stage. Non-CLR nomogram: harvested LNs + N stage

Moreover, the calibration curves demonstrated that the predicted survival rates calculated by the nomogram were in satisfying agreement with actual values, which implied a good consistency between prediction and actual observation (Fig. 3). ROC analyses were further applied to assess the discrimination of the nomogram, as shown in Fig. 4. The AUC in predicting 3-year OS was 0.77 (95% CI: 0.677−0.863) for the nomogram, 0.70 (95% CI: 0.604−0.805) for the non-CLR nomogram, and 0.67 (95% CI: 0.586−0.757) for TNM stage. Similarly, the three models also showed an obvious discrepancy in the prediction of 5-year OS of CRC patients, as reflected by the AUC of 0.74 (95% CI: 0.622−0.854) for the nomogram, 0.68 (95% CI: 0.561−0.807) for the non-CLR nomogram, and 0.71 (95% CI: 0.614−0.813) for TNM stage. The time-dependent ROC analysis indicated that the nomogram had the optimal survival discrimination during the time period from 12 to 60 months, in comparison with the non-CLR nomogram and TNM stage.

### Risk stratification of patients based on the nomogram

**Fig. 5:**
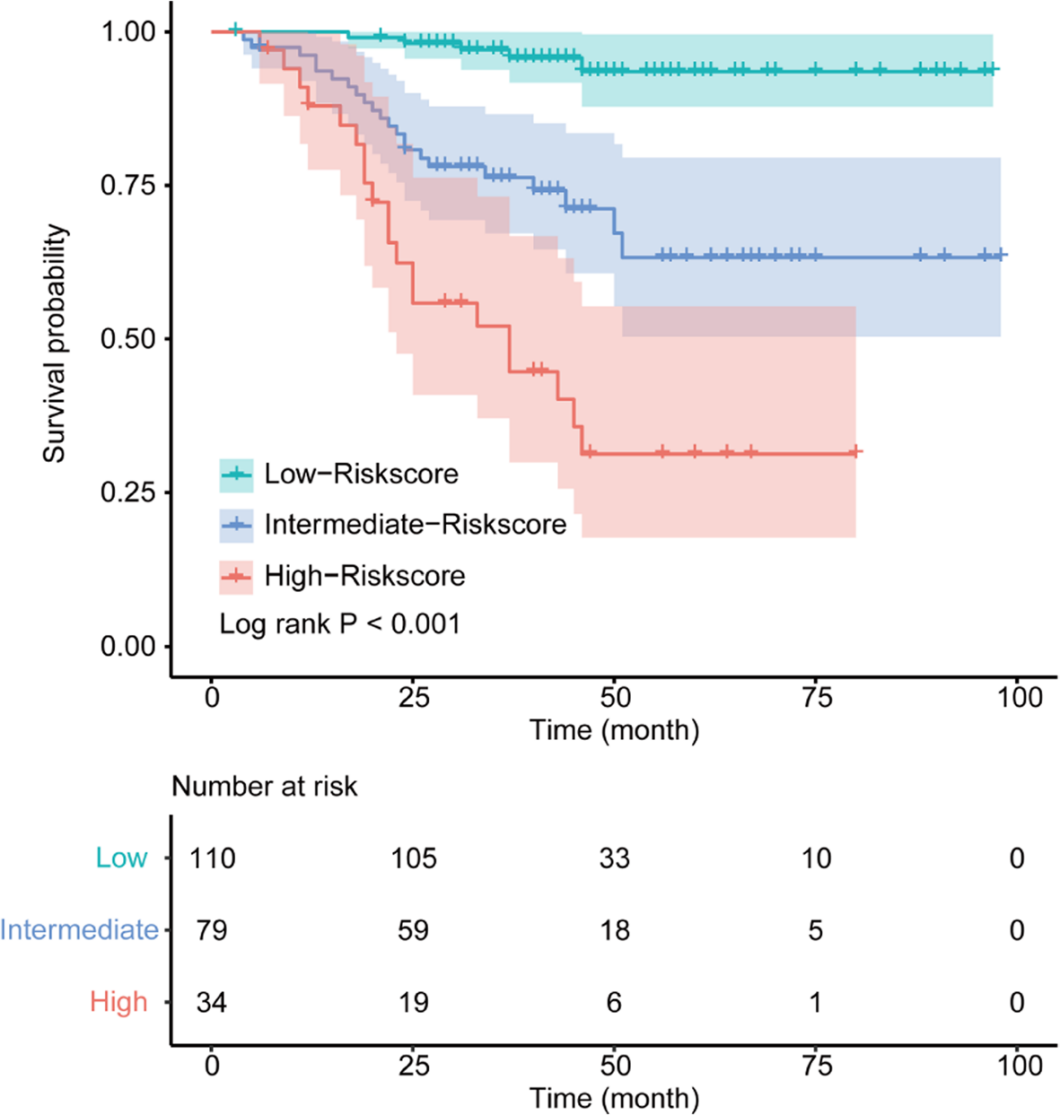
Survival curves for overall survival (OS) of colorectal cancer (CRC) patients stratified according to the total risk score which was obtained from the nomogram

Total risk scores of all 223 CRC patients were obtained based on the prognostic nomogram. After arranging all risk scores in ascending order, we set points at 50% and 85% percentiles as the cut-off values to categorize all patients into 3 levels (low, intermediate, high). The Kaplan-Meier analysis demonstrated that patients of different risk stratifications experienced significantly different OS (*P* < 0.001), as presented in Fig. 5.

## Discussion

In our study, 40% of the patients showed an elevation in CLR, and it was found that the elevated CLR was significantly associated with a decreased OS rate in CRC patients. Our study firstly described the correlation between CLR and CRC survival. Based on CLR and other clinical characteristics, we developed a nomogram that could give an accurate prediction for prognosis (Fig. 2). The prognostic nomogram showed favorable calibration and superior predictive performance than the TNM staging system and the non-CLR model, according to the *C*-index (Table 3) and ROC analysis (Fig. 4). Therefore, preoperative CLR was a novel biomarker that efficiently predicted the postoperative prognosis of CRC patients, and the nomogram could serve as a supplement to the TNM staging system, to assist clinicians in more accurate risk stratification of CRC patients.

Lipid disequilibrium and systemic inflammation played a vital role in tumor occurrence and development, immune escape, and metabolic pathway changes of CRC [36–40]. Several systemic inflammation-associated markers and serum lipid indexes were analyzed and used to predict the prognosis of CRC in previous studies. Although it was known that elevated cholesterol contributed to the prevalence of colorectal adenomas and tumors [41], conclusions of the relationship between serum cholesterol levels and the prognosis of CRC were inconsistent. Nielsen et al. found that a statin-induced reduction of serum cholesterol was capable of inhibiting tumor growth and metastasis and reducing the risk of mortality [42]. But another study showed no significant association between the decreased plasma cholesterol levels induced by statin and recurrence nor survival in stage III colon cancer patients [43]. In our study, cholesterol alone did not have predictive value for survival, but CLR, an indicator for the combination of cholesterol metabolism and systemic inflammation status, could well distinguish CRC patients with poor outcomes (Fig. 1). In addition, CLR was an independent prognostic indicator for OS in CRC (Table 2) and showed more valuable prognostic value than NLR, which is one of the most widely used systemic inflammation-associated biomarkers, according to the comparison using *C*-index in Table 3. Besides, from the subgroup analysis, we concluded that the effect of CLR on OS was more significant in stage III patients than stages I and II patients (Fig. 1). One of the possible reasons for this was that the survival rate was particularly high in cases without lymph node metastasis in our cohort (3-year OS rate: 0.908; 5-year OS rate: 0.863).

Tumor microenvironment (TME) received much attention in recent cancer literature, with a particular focus on its effect on tumor development and progression [44, 45]. Cholesterol metabolism and metabolite had significant roles in the TME, not only promoting cancer progressions, including oncocyte proliferation, migration, and invasion [34, 36–38], but also regulating inflammatory responses and innate immunity [46]. Cholesterol accumulation was shown to augment the pro-inflammation effect via the NF-κB signaling pathway in liver cancer cells [47]. Besides, the accumulation of cholesterol was also reported to accelerate the exhaustion of T cells, while functional tumor-infiltrating T lymphocytes serve an important role in anti-tumor immunity and positively correlated with the prognosis of CRC [48, 49]. Additionally, several studies indicated that the enrichment of oxysterols inhibited the anti-tumor effect of CD8 T cells by activating LXR signaling, and 27-hydroxycholesterol decreased CD8 T cells and increased neutrophils in the TME [50, 51]. Thus, it was worthwhile to consider the synergistic effect of cholesterol metabolism and immunosuppression on CRC prognosis simultaneously. And our results demonstrated the predictive value of CLR and incorporated it into the prognostic model. The model was expected to provide a novel idea to optimize the risk stratification for CRC patients.

However, there were several inevitable limitations. Firstly, this was a single-center retrospective design with 223 samples, which means our conclusion was based on the data of a relatively small sample size. Secondly, selection bias was present in this study. Since biochemical examination for blood lipids was not routinely performed in our center, a large number of patients who did not receive lipid measurements were not recruited. Thus, it was important to verify the results of our study in a large-scale prospective multi-center study.

## Conclusions

In conclusion, elevated CLR was a strong prognostic indicator for poor survival in CRC patients. The nomogram developed with CLR, harvested LNs, and N stage had satisfactory discrimination, calibration, and predictive accuracy in predicting the OS rate of patients with CRC. And a web-based dynamic nomogram (https://zhousiyu.shinyapps.io/CLRNomogram/) was further built to facilitate the prediction procedure. This study provided a potentially useful tool for clinicians to optimize the risk stratification and individualized treatment regimens for patients with CRC. And external cohorts are warranted to validate the clinical significance of the CLR-based model.

Authors’ contributions

Siyu Zhou and Zhigang Wang designed the study. Qian He, Jianfeng Gong, and Jiazi Ren collected the data and were major contributors in writing the manuscript. All the authors read and approved the final manuscript.

## Supplementary Information

**Additional file 1.**

**Additional file 2.**

## Data Availability

The data that support the findings of this study are available on request from the corresponding author.

## Abbreviations

CRC: Colorectal cancer
OS: Overall survival
LNs: Lymph nodes
CEA: Carcinoembryonic antigen
NLR: Neutrophil-to-lymphocyte ratio
CLR: Cholesterol-to-lymphocyte ratio
GPS: Glasgow prognostic score
mGPS: Modified Glasgow prognostic score
LMR: Lymphocyte-to-monocyte ratio
PLR: Platelet-to-lymphocyte ratio
*C*-index: Concordance index
CI: Confidence interval
ROC: Receiver operator characteristic curve
AUC: Area under the curve
KM: Kaplan-Meier
HR: Hazard ratio
TME: Tumor microenvironment
AJCC: American Joint Committee on Cancer

## References

[1] Erratum: Global cancer statistics 2018 (2020). GLOBOCAN estimates of incidence and mortality worldwide for 36 cancers in 185 countries. CA Cancer J Clin.

[2] Dekker E, Tanis PJ, Vleugels JLA, Kasi PM, Wallace MB (2019). Colorectal cancer. Lancet.

[3] Wang W, Kandimalla R, Huang H, Zhu L, Li Y, Gao F, Goel A, Wang X (2019). Molecular subtyping of colorectal cancer: recent progress, new challenges and emerging opportunities. Semin Cancer Biol.

[4] Coppedè F, Lopomo A, Spisni R, Migliore L (2014). Genetic and epigenetic biomarkers for diagnosis, prognosis and treatment of colorectal cancer. World J Gastroenterol.

[5] Sagaert X, Vanstapel A, Verbeek S (2018). Tumor heterogeneity in colorectal cancer: what do we know so far?. Pathobiology.

[6] Liebig C, Ayala G, Wilks J, Verstovsek G, Liu H, Agarwal N, Berger DH, Albo D (2009). Perineural invasion is an independent predictor of outcome in colorectal cancer. J Clin Oncol.

[7] Cho YB, Chun HK, Yun HR, Kim HC, Yun SH, Lee WY (2009). Histological grade predicts survival time associated with recurrence after resection for colorectal cancer. Hepatogastroenterology.

[8] Liu X, Liang Y, Song R, Yang G, Han J, Lan Y, Pan S, Zhu M, Liu Y, Wang Y (2018). Long non-coding RNA NEAT1-modulated abnormal lipolysis via ATGL drives hepatocellular carcinoma proliferation. Mol Cancer.

[9] Huang B, Song BL, Xu C (2020). Cholesterol metabolism in cancer: mechanisms and therapeutic opportunities. Nat Metab.

[10] Zeljkovic A, Vekic J, Mihajlovic M, Gojkovic T, Vladimirov S, Zeljkovic D, Spasojevic-Kalimanovska V, Trifunovic B (2021). Revealing the role of high-density lipoprotein in colorectal cancer. Int J Mol Sci.

[11] Long J, Zhang CJ, Zhu N, Du K, Yin YF, Tan X, Liao DF, Qin L (2018). Lipid metabolism and carcinogenesis, cancer development. Am J Cancer Res.

[12] Broitman SA, Cerda S (1993). Wilkinson Jt: Cholesterol metabolism and colon cancer. Prog Food Nutr Sci.

[13] Ma XL, Gao XH, Gong ZJ, Wu J, Tian L, Zhang CY, Zhou Y, Sun YF, Hu B, Qiu SJ (2016). Apolipoprotein A1: a novel serum biomarker for predicting the prognosis of hepatocellular carcinoma after curative resection. Oncotarget.

[14] Sirniö P, Väyrynen JP, Klintrup K, Mäkelä J, Mäkinen MJ, Karttunen TJ, Tuomisto A (2017). Decreased serum apolipoprotein A1 levels are associated with poor survival and systemic inflammatory response in colorectal cancer. Sci Rep.

[15] van Duijnhoven FJ, Bueno-De-Mesquita HB, Calligaro M, Jenab M, Pischon T, Jansen EH, Frohlich J, Ayyobi A, Overvad K, Toft-Petersen AP (2011). Blood lipid and lipoprotein concentrations and colorectal cancer risk in the European prospective investigation into cancer and nutrition. Gut.

[16] Kitahara CM, Berrington de González A, Freedman ND, Huxley R, Mok Y, Jee SH, Samet JM (2011). Total cholesterol and cancer risk in a large prospective study in Korea. J Clin Oncol.

[17] Iftimie S, Escribano A, Díez-Sans A, Albiciuc I, Hernández-Aguilera A, Fort-Gallifa I, López-Azcona AF, Camps J, Joven J, Castro A (2021). Influence of surgical procedures on serum paraoxonase-1-related variables and markers of inflammation in hospitalized patients. J Invest Surg.

[18] Coussens LM, Werb Z (2002). Inflammation and cancer. Nature.

[19] Rossi S, Basso M, Strippoli A, Schinzari G, D'Argento E, Larocca M, Cassano A, Barone C (2017). Are markers of systemic inflammation good prognostic indicators in colorectal cancer?. Clin Colorectal Cancer.

[20] Ikeguchi M, Urushibara S, Shimoda R, Yamamoto M, Maeta Y, Ashida K (2014). Inflammation-based prognostic scores and nutritional prognostic index in patients with locally-advanced unresectable colorectal cancer. World J Surg Oncol.

[21] Paliogiannis P, Deidda S, Maslyankov S, Paycheva T, Farag A, Mashhour A, Misiakos E, Papakonstantinou D, Mik M, Losinska J (2020). Blood cell count indexes as predictors of anastomotic leakage in elective colorectal surgery: a multicenter study on 1432 patients. World J Surg Oncol.

[22] Alsaif SH, Rogers AC, Pua P, Casey PT, Aherne GG, Brannigan AE, Mulsow JJ, Shields CJ, Cahill RA (2021). Preoperative C-reactive protein and other inflammatory markers as predictors of postoperative complications in patients with colorectal neoplasia. World J Surg Oncol.

[23] Wu Y, Li C, Zhao J, Yang L, Liu F, Zheng H, Wang Z, Xu Y (2016). Neutrophil-to-lymphocyte and platelet-to-lymphocyte ratios predict chemotherapy outcomes and prognosis in patients with colorectal cancer and synchronous liver metastasis. World J Surg Oncol.

[24] Huang X, Cui J, Li X, Liu C, Sun J, Yue J (2021). The decreased platelet-to-lymphocyte ratio could predict a good prognosis in patients with oligometastatic colorectal cancer: a single-center cohort retrospective study. World J Surg Oncol.

[25] Liu Q, Luo D, Cai S, Li Q, Li X (2020). Circulating basophil count as a prognostic marker of tumor aggressiveness and survival outcomes in colorectal cancer. Clin Transl Med.

[26] Schwartz PB, Poultsides G, Roggin K, Howard JH, Fields RC, Clarke CN, Votanopoulos K, Cardona K, Winslow ER (2020). PLR and NLR are poor predictors of survival outcomes in sarcomas: a new perspective from the USSC. J Surg Res.

[27] Seetohul YB, Singh V, Jain RK, Chaudhary AK (2020). Prognostic value of neutrophil-lymphocyte ratio and platelet-lymphocyte ratio in head and neck malignancies. Indian J Otolaryngol Head Neck Surg.

[28] Xia LJ, Li W, Zhai JC, Yan CW, Chen JB, Yang H (2020). Significance of neutrophil-to-lymphocyte ratio, platelet-to-lymphocyte ratio, lymphocyte-to-monocyte ratio and prognostic nutritional index for predicting clinical outcomes in T1-2 rectal cancer. BMC Cancer.

[29] Lu J, Xu BB, Zheng ZF, Xie JW, Wang JB, Lin JX, Chen QY, Cao LL, Lin M, Tu RH (2019). CRP/prealbumin, a novel inflammatory index for predicting recurrence after radical resection in gastric cancer patients: post hoc analysis of a randomized phase III trial. Gastric Cancer.

[30] Chen JH, Zhai ET, Yuan YJ, Wu KM, Xu JB, Peng JJ, Chen CQ, He YL, Cai SR (2017). Systemic immune-inflammation index for predicting prognosis of colorectal cancer. World J Gastroenterol.

[31] Liao CK, Yu YL, Lin YC, Hsu YJ, Chern YJ, Chiang JM, You JF (2021). Prognostic value of the C-reactive protein to albumin ratio in colorectal cancer: an updated systematic review and meta-analysis. World J Surg Oncol.

[32] Xu H, You G, Zhang M, Song T, Zhang H, Yang J, Jia Y, Tang J, Liang X (2019). Association of pre-surgery to pre-radiotherapy lymphocyte counts ratio with disease-free survival in rectal cancer patients receiving neoadjuvant concurrent chemoradiotherapy. World J Surg Oncol.

[33] Wang Y, Sun XQ, Lin HC, Wang DS, Wang ZQ, Shao Q, Wang FH, Yan SM, Liang JY, Zeng ZL (2019). Correlation between immune signature and high-density lipoprotein cholesterol level in stage II/III colorectal cancer. Cancer Med.

[34] Zhang J, Zhao B, Jin F (2019). The assessment of 8th edition AJCC prognostic staging system and a simplified staging system for breast cancer: the analytic results from the SEER database. Breast J.

[35] Liao F, He W, Jiang C, Yin C, Guo G, Chen X, Qiu H, Rong Y, Zhang B, Xu D, Xia L (2015). A high LDL-C to HDL-C ratio predicts poor prognosis for initially metastatic colorectal cancer patients with elevations in LDL-C. Onco Targets Ther.

[36] Chimento A, Casaburi I, Avena P, Trotta F, De Luca A, Rago V, Pezzi V, Sirianni R (2018). Cholesterol and its metabolites in tumor growth: therapeutic potential of statins in cancer treatment. Front Endocrinol (Lausanne).

[37] Ding X, Zhang W, Li S, Yang H (2019). The role of cholesterol metabolism in cancer. Am J Cancer Res.

[38] Wang Y, Liu C, Hu L (2019). Cholesterol regulates cell proliferation and apoptosis of colorectal cancer by modulating miR-33a-PIM3 pathway. Biochem Biophys Res Commun.

[39] Liu Z, Liu X, Liu S, Cao Q (2018). Cholesterol promotes the migration and invasion of renal carcinoma cells by regulating the KLF5/miR-27a/FBXW7 pathway. Biochem Biophys Res Commun.

[40] Tuomisto AE, Mäkinen MJ, Väyrynen JP (2019). Systemic inflammation in colorectal cancer: underlying factors, effects, and prognostic significance. World J Gastroenterol.

[41] Yang MH, Rampal S, Sung J, Choi YH, Son HJ, Lee JH, Kim YH, Chang DK, Rhee PL, Kim JJ (2013). The association of serum lipids with colorectal adenomas. Am J Gastroenterol.

[42] Nielsen SF, Nordestgaard BG, Bojesen SE (2012). Statin use and reduced cancer-related mortality. N Engl J Med.

[43] Ng K, Ogino S, Meyerhardt JA, Chan JA, Chan AT, Niedzwiecki D, Hollis D, Saltz LB, Mayer RJ, Benson AB (2011). Relationship between statin use and colon cancer recurrence and survival: results from CALGB 89803. J Natl Cancer Inst.

[44] Xu H, Zhou S, Tang Q, Xia H, Bi F (2020). Cholesterol metabolism: new functions and therapeutic approaches in cancer. Biochim Biophys Acta Rev Cancer.

[45] Yang Y, Liu X, Ma W, Xu Q, Chen G, Wang Y, Xiao H, Li N, Liang XJ, Yu M, Yu Z (2021). Light-activatable liposomes for repetitive on-demand drug release and immunopotentiation in hypoxic tumor therapy. Biomaterials.

[46] Tall AR, Yvan-Charvet L (2015). Cholesterol, inflammation and innate immunity. Nat Rev Immunol.

[47] He M, Zhang W, Dong Y, Wang L, Fang T, Tang W, Lv B, Chen G, Yang B, Huang P, Xia J (2017). Pro-inflammation NF-κB signaling triggers a positive feedback via enhancing cholesterol accumulation in liver cancer cells. J Exp Clin Cancer Res.

[48] Ma X, Bi E, Lu Y, Su P, Huang C, Liu L, Wang Q, Yang M, Kalady MF, Qian J (2019). Cholesterol induces CD8(+) T cell exhaustion in the tumor microenvironment. Cell Metab.

[49] Zhao Y, Ge X, He J, Cheng Y, Wang Z, Wang J, Sun L (2019). The prognostic value of tumor-infiltrating lymphocytes in colorectal cancer differs by anatomical subsite: a systematic review and meta-analysis. World J Surg Oncol.

[50] Raccosta L, Fontana R, Maggioni D, Lanterna C, Villablanca EJ, Paniccia A, Musumeci A, Chiricozzi E, Trincavelli ML, Daniele S (2013). The oxysterol-CXCR2 axis plays a key role in the recruitment of tumor-promoting neutrophils. J Exp Med.

[51] Bensinger SJ, Bradley MN, Joseph SB, Zelcer N, Janssen EM, Hausner MA, Shih R, Parks JS, Edwards PA, Jamieson BD, Tontonoz P (2008). LXR signaling couples sterol metabolism to proliferation in the acquired immune response. Cell.

