# Supplementary material for: Blood cholesterol-to-lymphocyte ratio as a novel prognostic marker to predict postoperative overall survival in patients with colorectal cancer
Source: World J Surg Oncol. 2022 Jan 15;20:18. doi: 10.1186/s12957-021-02471-4 (PMC8760814; doi:10.1186/s12957-021-02471-4)
Supplement: Supplementary file 1 — Additional file 1. [file 12957_2021_2471_MOESM1_ESM.docx]

**Supplementary Table 1.** The final prognostic model in the Cox hazard proportion regression.

| Variables | Coef | SE | Z | HR | 95% CI | P value |
| --- | --- | --- | --- | --- | --- | --- |
| pN stage |  |  |  |  |  |  |
| N1 vs. N0 | 1.332 | 0.343 | 3.885 | 3.789 | 1.935-7.42 | <0.001 |
| N2 vs. N0 | 1.668 | 0.411 | 4.058 | 5.303 | 2.369-11.871 | <0.001 |
| Harvested LNs | -0.053 | 0.025 | -2.114 | 0.948 | 0.903-0.996 | 0.035 |
| CLR (high vs. low) | 1.107 | 0.303 | 3.656 | 3.024 | 1.671-5.473 | <0.001 |

**Abbreviations:** Coef, coefficient; SE, standard error; HR, hazard ratio; CI, confidence interval.
