# Supplementary material for: Blood cholesterol-to-lymphocyte ratio as a novel prognostic marker to predict postoperative overall survival in patients with colorectal cancer
Source: World J Surg Oncol. 2022 Jan 15;20:18. doi: 10.1186/s12957-021-02471-4 (PMC8760814; doi:10.1186/s12957-021-02471-4)
Supplement: Supplementary file 2 — Additional file 2. [file 12957_2021_2471_MOESM2_ESM.docx]

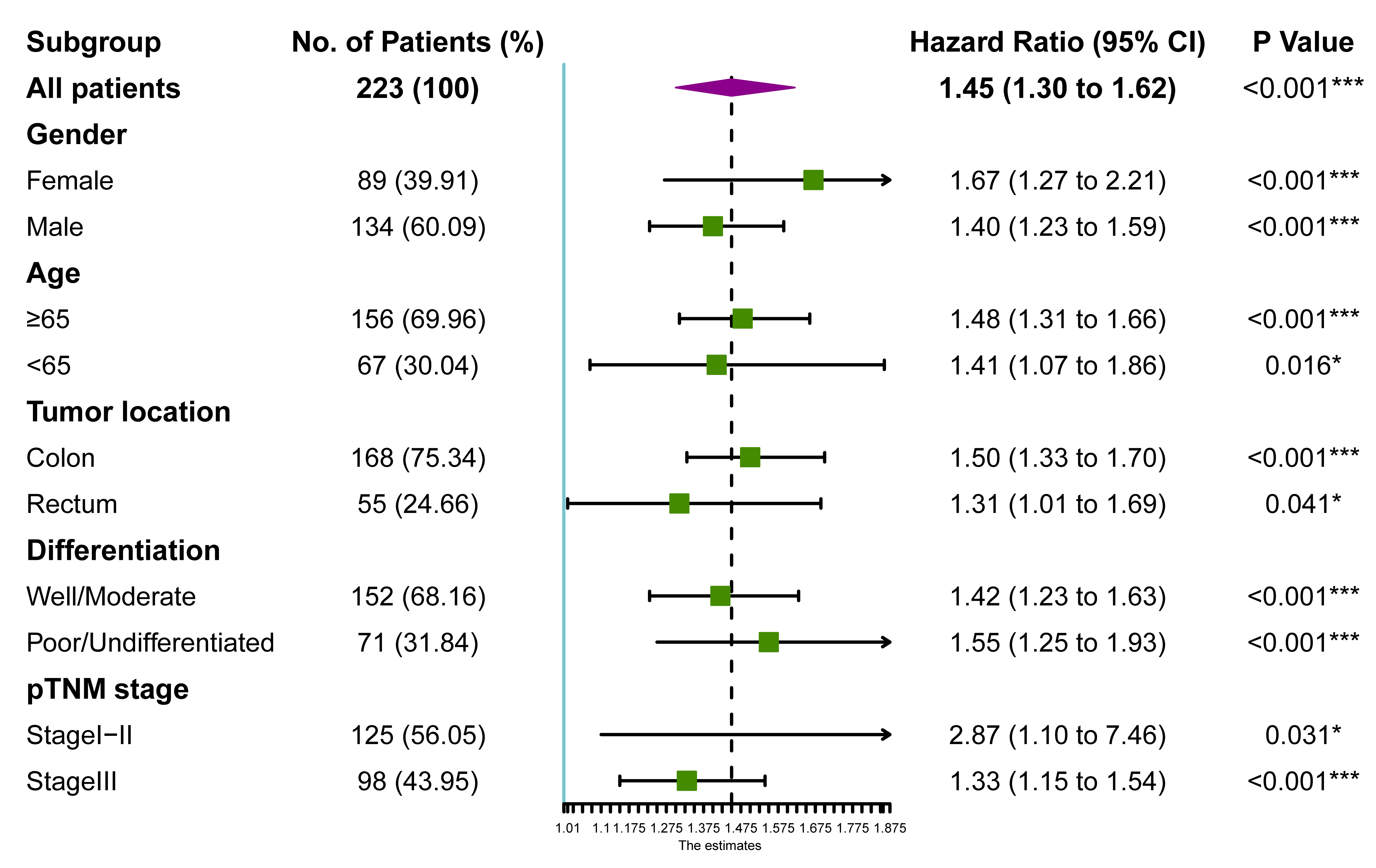


**Supplementary Fig. 1** Association of nomogram score with overall survival in all colorectal cancer (CRC) patients and subgroups of patients with different ages, genders, tumor locations, tumor differentiation, and clinical stages. CI, confidence interval.
